# Role of necroptosis in airflow limitation in chronic obstructive pulmonary disease: focus on small-airway disease and emphysema

**DOI:** 10.1038/s41420-022-01154-7

**Published:** 2022-08-16

**Authors:** Chanjing Liu, Peijun Li, Jiejiao Zheng, Yingqi Wang, Weibing Wu, Xiaodan Liu

**Affiliations:** 1 Department of Sports Rehabilitation, Shanghai University of Sport, Shanghai, People’s Republic of China; 2 Department of Rehabilitation Medicine, Huadong Hospital, Shanghai, People’s Republic of China; 3 School of Rehabilitation Science, Shanghai University of Traditional Chinese Medicine, Shanghai, People’s Republic of China

**Keywords:** Chronic obstructive pulmonary disease, Necroptosis

## Abstract

Airflow limitation with intractable progressive mechanisms is the main disease feature of chronic obstructive pulmonary disease (COPD). The pathological process of airflow limitation in COPD involves necroptosis, a form of programmed necrotic cell death with pro-inflammatory properties. In this paper, the correlations of small-airway disease and emphysema with airflow limitation in COPD were firstly reviewed; then, based on this, the effects of necroptosis on small-airway disease and emphysema were analysed, and the possible mechanisms of necroptosis causing airflow limitation in COPD were explored. The results showed that airflow limitation is caused by a combination of small-airway disease and emphysema. In addition, toxic particulate matter stimulates epithelial cells to trigger necroptosis, and necroptosis promotes the expulsion of cell contents, the abnormal hyperplasia of pro-inflammatory mediators and the insufficient clearance of dead cells by macrophages; these processes, coupled with the interaction of necroptosis and oxidative stress, collectively result in small-airway disease and emphysema in COPD.

## Facts

1. Necroptosis induces small-airway disease and acts as an emphysema-specific event rather than apoptosis.
2. Necroptosis is implicated in the pathophysiology of chronic obstructive pulmonary disease (COPD) through various signalling pathways.
3. A complex link exists between necroptosis and apoptosis in COPD.

## Open questions

1. Can necroptosis be ultimately targeted for therapeutic interventions in airflow limitation in COPD?
2. Can the transformation between apoptosis and necroptosis be artificially controlled?

## Introduction

Chronic obstructive pulmonary disease (COPD) is the third leading cause of death globally [1]. The Global Initiative for Chronic Obstructive Lung Disease clearly states that COPD is characterised by expiratory airflow limitation resulting from increased resistance due to small-airway disease and increased lung compliance caused by parenchymal destruction (emphysema) [2]. The degree of airflow limitation shows progressive exacerbation. Moreover, airway inflammation and emphysema, as the causes of airflow limitation, continue to worsen years after smoking cessation [3–5], indicating the intractable progressive mechanisms of airflow limitation in COPD. Improvements in the progressive mechanisms of airflow limitation can help with optimising clinical treatment.

Currently, bronchodilators (including β-agonists, theophylline and anti-cholinergic drugs) and anti-inflammatory drugs are the medications most commonly used to relieve restricted expiratory symptoms in patients with COPD and act by relaxing the bronchial smooth muscle or inhibiting inflammation. These drugs can delay the decline of lung function, facilitate more effective lung-emptying, remarkably improve movement tolerance and relieve dyspnoea, but they cannot prevent the progression of airflow restriction [6, 7]. Therefore, further exploration of the intractable progression of airflow limitation in COPD is necessary to find a more accurate or complete therapeutic target.

Necroptosis occurs after cell stress or injury and leads to necrotic morphology and the release of cell contents, inducing an inflammatory response. Necroptosis under normal circumstances is used to maintain homeostasis, but excess or deficiency can cause adverse effects, such as small-airway disease and emphysema. A recent study by Lu et al. [8] showed that necroptosis is considerably increased in the lungs of patients with COPD and that inhibition of necroptosis can alleviate airway inflammation, airway remodelling and emphysema in mouse models of COPD induced by cigarette smoke (CS) exposure. The results of particulate matter (PM) intervention in human bronchial epithelial cells to mimic pathological processes in vitro also showed that necroptosis inhibitors can reduce inflammatory cytokines and mucin release [9]. Thus, necroptosis is a potential therapeutic target for preventing the progressive exacerbation of airflow limitation in COPD. Therefore, this paper reviews the role of necroptosis in airflow limitation in COPD, further discusses the related mechanisms to clarify the possible pathology of airflow limitation in COPD and provides a theoretical basis for clinical treatment.

## Airflow limitation in COPD

The respiratory process is divided into pulmonary ventilation and gas exchange. Pulmonary ventilation involves the passage of air in and out of the airways and alveoli. Maintaining adequate airflow during this period is the basis for normal breathing; otherwise, airflow limitation occurs. The main feature of COPD is the slow, progressive and irreversible expiratory airflow limitation. The Global Initiative for Chronic Obstructive Lung Disease uses the degree of airway limitation for COPD diagnosis and the staging of disease severity. Common indicators include forced expiratory volume in 1 second (FEV1) and the ratio of FEV1 to forced vital capacity (FVC) [2]. The pathological changes that cause airflow limitation in COPD include small-airway disease and emphysema.

### Small-airway disease induces airflow limitation

The airway acts as a passage for airflow. It branches gradually from the carina to the distal terminal bronchioles (alveoli). Airway epithelial barriers as innate defence systems are disrupted by direct exposure to toxic particles, causing increased permeability and the release of large amounts of inflammatory cytokines and mucus. In COPD progression, the airway is blocked by inflammatory exudates containing mucus, particularly in small conducting airways (<2 mm in diameter) [10]. A decrease in FEV1 is related to increased airway infiltration by inflammatory cells, such as macrophages, CD4^+^ T and CD8^+^ T lymphocytes and B lymphocytes [11, 12]. Besides having increased inflammatory cells, goblet cells are also increased in the small-airway epithelium in COPD and are closely related to FEV1/FVC [13]. Increased goblet cells can alter the normal surfactant in the airway fluid membrane by producing excess mucus, making the small airways more easily close at low lung volumes [14]. Excess mucus can also lead to small-airway obstruction and affect normal ventilation function. In addition, increased thickness of the airway wall, which results from the thickened airway epithelium, outer membrane and bronchial smooth muscle, in patients with COPD reduces the calibre of airflow passages, which leads to the same degree of smooth muscle shortening, triggers greater lumen stenosis and contributes to airway hyper-responsiveness under non-specific stimulation in patients with COPD; furthermore, thickening of the airway wall is negatively associated with FEV1 [12, 15]. Thus, small-airway disease (including airway inflammation, mucus overproduction and airway remodelling) in COPD causes lumen stenosis, which leads to a substantial increase in airway resistance and ultimately expiratory airflow limitation.

### Emphysema induces airflow limitation

The lungs are elastic. The potential energy generated by diaphragm contraction during inspiration is stored in elastic tissues and forms the elastic recoil of the lungs, which is released to constitute the impetus of lung-emptying during exhalation. The pathological changes in emphysema, a common manifestation of COPD, include irreversible destruction of alveolar walls, excessive dilation of the distal terminal bronchioles and reduced elastic recoil of the lung caused by disruption of the lung parenchyma. Elastic recoil measured at 90% of the total lung volume is associated with emphysema and FEV1 in patients with COPD, indicating that the loss of elastic recoil due to emphysema is one of the determinants of airflow limitation [16]. Emphysema also reduces the elastic load imposed on the airway by destroying alveolar attachments (i.e. the alveolar walls directly attached to the airway), which makes it difficult to keep the airway open, especially at low lung volumes [17]. In addition, static/dynamic lung hyperinflation due to emphysema can cause thoracic deformation, in which the diaphragm is positioned abnormally within the thorax, increasing its mechanical load and resulting in insufficient diaphragm retraction on exhalation. Diaphragm mobility is also related to FEV1 [18]. In conclusion, emphysema leads to reduced lung elastic recoil, airway elastic load and diaphragm mobility, resulting in expiratory airflow limitation.

The product of airway resistance and lung compliance provides the time constant for lung-emptying that is reflected in FEV1 and FEV1/FVC measurements, whereas airflow limitation is the result of the prolonged time constant of lung-emptying [19]. Mao et al. [20] confirmed that serum melatonin levels positively correlate with FEV1 and FEV1/FVC in acute COPD and found that melatonin exerts a lung-protective effect via the MT1/MT2 membrane receptor to inhibit the necroptosis pathway in COPD mouse models. Thus, necroptosis involves small-airway disease and emphysema, leading to airflow limitation in COPD.

## Necroptosis

Necroptosis is the programmed mode of active cell necrosis characterised morphologically by organelle swelling, plasma membrane rupture and cell lysis. When caspase (an apoptosis executive protein) is absent or inhibited, death receptors and Toll-like receptors on the cell membrane connect with extracellular ligands (e.g. tumour necrosis factor) to send an activation signal; then, receptor-interacting protein kinase 1 (RIPK1) activated by this signal phosphorylates RIPK3, and RIPK1 and RIPK3 together form an intracellular amyloid-like complex to assemble the necrosome, which acts as the necroptosis signalling transducer [21]. RIPK3 mediates the phosphorylation of mixed-lineage kinase domain-like (MLKL, a necroptosis executive protein), which results in MLKL oligomerisation and translocation to the inner leaflet of the plasma membrane for forming plasma membrane pores; then, plasma membrane rupture and leakage, which eventually leads to cell death [22]. In addition, DNA-dependent activators of interferon regulatory factors and Toll-like receptors that use TIR-domain-containing adapter-inducing interferon-β, as trigger factors in necroptotic cells, can directly activate RIPK3 to induce necroptosis by detecting exogenous viral nucleic acids [23, 24]. Chen et al. [25] also confirmed that RIPK3/MLKL has a direct pathway of activation in necroptosis induced by CS extract (CSE) through CSE-exposed epithelial cells treated with different protein inhibitors and gene-knockout methods.

**Fig. 1:**
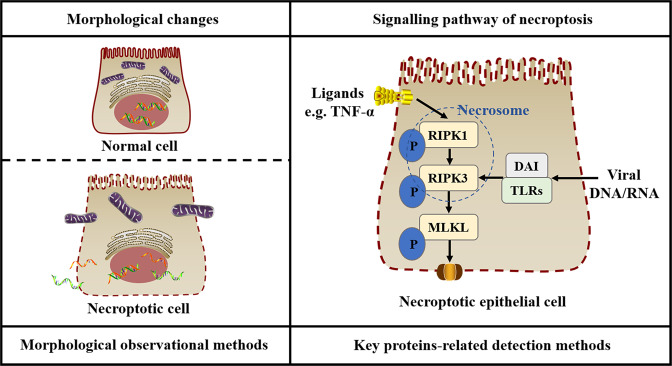
Main detection methods of necroptosis. The left figure is a schematic diagram of the morphological changes in the necroptotic cell. The figure shows cellular swelling, organelle swelling, plasma membrane rupture, and cell lysis causing the release of the contents. Generally, it can be observed directly by an electron microscope. The right figure is a schematic diagram of the necroptotic signalling pathway. The figure shows RIPK1 phosphorylation upon receiving a signal from ligand binding and then RIPK3 activation, which interacts with RIPK1 to form a necrosome. Phosphorylated MLKL mediated by RIPK3 induces the formation of plasma membrane pores. In addition, DAI and TLRs can directly activate RIPK3 leading to necroptosis after detecting exogenous viral DNA or RNA. Key proteins in the necroptotic signalling pathway, such as RIPK3 and MLKL, can be detected by Western Blot, Immunohistochemistry or Enzyme-Linked Immunosorbent Assay. The expression levels of the genes corresponding to key proteins can be detected by Polymerase Chain Reaction. The above methods are suitable for detecting necroptosis. DAI DNA-dependent activator of interferon regulatory factors, MLKL mixed-lineage kinase domain-like, P phosphorylates, RIPK receptor-interacting protein kinase, TLRs toll-like receptors, TNF tumour necrosis factor.

Necroptosis is morphologically distinct from other cell death modes except for necrosis and is heavily dependent on RIPK3 and MLKL in terms of pathways, which suggests the main detection methods of necroptosis, as shown in Fig. 1. The common methods currently used to distinguish necroptosis from other cell death modes include the direct observation of cell death morphology, the detection of RIPK3/MLKL protein expression level and activation degree and the comparison between the control group and the experimental group treated with the key protein inhibitors of the pathway or related gene-knockout methods. Most previous studies chose TUNEL staining to determine the mode of cell death by assessing DNA breakage, in which a positive result indicates apoptosis. However, DNA fragment breaks are not a specific result of apoptosis; the detection can also identify cells that are undergoing necroptosis or necrosis [26]. Therefore, the non-specificity of the detection may be one of the reasons why the effect of necroptosis on airflow limitation in COPD is still not elucidated.

A recent study applied necroptosis-specific detection and found that patients with COPD have remarkably increased phosphorylated RIPK3 (p-RIPK3) and p-MLKL in lung tissue homogenates relative to those without airflow limitation; furthermore, the differences remained substantial after adjusting for age, gender and current smoking status of patients [8]. This study indicated that necroptosis is considerably induced in the lungs of patients with COPD and that necroptosis and airflow limitation in COPD are strongly associated.

## Necroptosis induces airflow limitation in COPD

### Necroptosis induces small-airway disease

Necroptosis induced by multiple common stimuli for COPD models promotes an abnormal increase in the inflammatory response and mucin levels, leading to airway inflammation, mucus overproduction and airway remodelling. Different types of epithelial cells (e.g. human bronchial epithelial cells, mouse lung epithelial cells [MLE]-12, human normal lung epithelial cells) were exposed to CSE or PM to simulate the lung epithelia of patients with COPD who were directly exposed to toxic PM, and treatment with Nec-1 (a RIPK1 inhibitor)/GSK'872 (a RIPK3 inhibitor)/theaflavin-3,3′-digallate (TF-3, which is proven to inhibit necroptosis) remarkably reduced the expression of inflammatory cytokines and mucins that make up the airway mucus. The results indicate that necroptosis promotes inflammatory responses and mucin production at the cellular level [9, 27, 28]. In addition, the inhibition of necroptosis of macrophages exposed to CSE can also prevent the production of inflammatory cytokines [29]. Epithelial cells were co-cultured with macrophages under CSE exposure, pre-treated with necroptosis or apoptosis inhibitors and detected for inflammation-related markers. The results revealed that CSE-induced necroptosis is pro-inflammatory and may lead to lung injury; meanwhile, apoptosis has either no effect or an inhibitory effect on inflammation, which was verified again as a conclusion in CS-exposed mice [25]. Xu et al. [9] detected inflammation and mucin in the airways of mice treated with PM and necroptosis inhibitors and found that necroptosis inhibition could reduce lung inflammation and the excessive secretion of airway mucus in mice—that is, necroptosis contributes to the pathogenesis of PM-induced lung injury, which suggests that necroptosis is closely related to airway inflammation and mucus overproduction in COPD. Pouwels et al. [30] systematically exposed mice to CS after the intraperitoneal injection of Nec-1 and found that Nec-1 pre-treatment remarkably inhibited CS-induced neutrophilic airway inflammation by assessing the number of neutrophils, the degree of activation and inflammatory markers in the bronchoalveolar lavage fluid of mice, confirming that necroptosis has a role in airway inflammation. Mao et al. [20] showed that melatonin alleviates inflammatory cell infiltration into the airway, reduces the airway wall thickness and increases the number of cilia in bronchial epithelial cells by inhibiting necroptosis, which suggests that necroptosis causes airway inflammation and airway remodelling in COPD based on a mouse model of COPD-like inflammation caused by nebulised inhaled lipopolysaccharide. RIPK3-deficient (^−/−^) and MLKL^−/−^ mice were compared to normal mice under acute and chronic CS exposure, and the results revealed a remarkable decrease in inflammatory cells, chemokines, airway basement membrane thickness and collagen deposition in lung tissues, confirming once again that necroptosis is an upstream mechanism leading to airway inflammation and airway remodelling [8].

### Necroptosis induces emphysema

Apoptosis has long been believed to play an important role in lung structural remodelling and tissue destruction in COPD, and the rate of positive TUNEL staining is remarkably increased in the emphysema region [31]. However, a recent study found that, although apoptosis and necroptosis can be induced by toxic PM, increased cellular stress causes a shift in the cell death mode first from early to late apoptosis and then eventually to necroptosis [25]. This result showed that the decrease in apoptotic epithelial cells is inconsistent with the trend of increased dead cells and exacerbated emphysema in the COPD process. In addition, Mouded et al. [32] induced apoptosis by microcystin in mouse endotracheal cells, while comparing the lungs of microcystin-treated and porcine pancreatic elastase-treated mice, where they found that apoptosis-induced alveolar enlargement had manifested as transient acute lung injury with alveolar collapse, fibrosis and adjacent alveolar enlargement but not irreversible emphysema. The above findings suggest that the cause of emphysema in COPD is likely to be necroptosis rather than apoptosis. In lipopolysaccharide- or CSE-induced mouse models, melatonin and TF-3 have been shown to alleviate lung inflammation, reduce the area of lung destruction and attenuate emphysematous changes by inhibiting necroptosis [20, 28]. Chen et al. [25] injected a necroptosis or apoptosis inhibitor before CS exposure and found that emphysema exacerbation can be slowed down by necroptosis inhibition but not by apoptosis inhibition according to the measured chord lengths of lungs and alveolar area in mice. Lu et al. [8] compared the extent of CS-induced emphysema in knockout and normal mice and found that RIPK3 and MLKL deletion are effective in alleviating emphysema development and matrix metalloproteinase 12 expression as a key molecule in emphysema. However, the alterations in matrix metalloproteinase 12 in RIPK3^−/−^ mice were not substantial, which suggests that MLKL deletion is more effective in preventing emphysema than RIPK3 deletion, possibly because RIPK3 affects apoptotic and necroptotic pathways, whereas MLKL represents a necroptosis-specific effect. In conclusion, emphysema is a product of necroptosis.

**Fig. 2:**
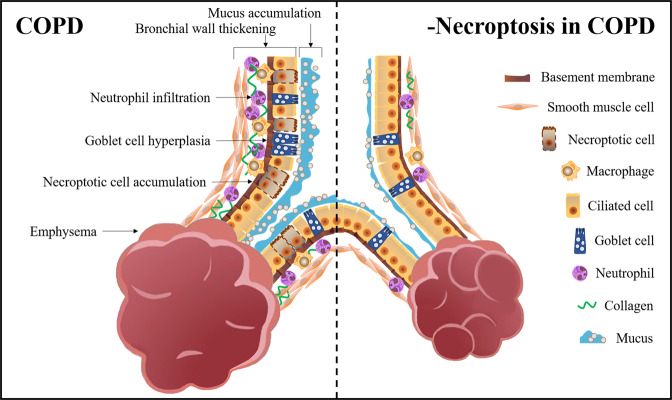
Pathological changes after inhibiting necroptosis in COPD. The left side of the dashed line shows the pathological manifestations of airflow limitation in COPD, including small-airway disease and emphysema; the right side shows the pathological changes after inhibiting necroptosis in COPD, the specific as follows: in the small airway, the increased number of ciliated cells, the reduced thickness of the bronchial wall, and the improvements of neutrophilic airway inflammation, mucus accumulation and collagen deposition; in the alveoli, the improvements of alveolar lumen enlargement and alveolar wall destruction.

Table 1 shows the related literature on necroptosis causing small-airway disease and emphysema in COPD. Figure 2 shows the pathological changes after inhibiting necroptosis in COPD.

**Table 1:** The role of necroptosis in small-airway disease and emphysema.

Study	Research objects	Group	Necroptosis indicators	Small-airway disease indicators	Emphysema indicators
Wang Y, 2018	HBE cells	EG: Nec-1 + CSE CG: DMSO + CSE	/	IL6, IL8, MUC5AC ↓	/
XU F, 2018	HBE cells	EG: Nec-1/GSK'872 + PM CG: DMSO + PM	PI-positive cells↓	IL6, IL8, MUC5AC ↓	/
	Male C57BL/6 mice (aged 6-8 weeks)	EG: Nec-1 + PM CG: DMSO + PM	/	Total inflammatory cells, neutrophils↓ MUC5AC, CXCL1, CXCL2, G-CSF ↓ Inflammation score↓	/
LUAN G, 2022	BEAS-2B cells	EG: TF-3 + CSE CG: CSE	/	TNF-α, IL-6, IL-1β ↓	/
	BEAS-2B cells	EG: TF-3 + CSE + z-VAD Nec-1 + CSE + z-VAD CG: CSE + z-VAD	p-RIPK3, p-MLKL ↓ PI-positive cells↓ Necroptotic rate↓	/	/
	Male C57BL/6 J mice (aged 7 weeks)	EG: TF-3 + CSE CG: CSE	p-RIPK3, p-MLKL ↓	TNF-α, IL-1β ↓	Emphysematous changes, MLI, DI ↓
Wang Y, 2020	BMDM cells	EG: Nec-1 + CSE GSK'872 + CSE CG: DMSO + CSE	/	CXCL1, CXCL2, IL-6↓	/
CHEN D, 2021	MLE-12 cells cocultured with BMDMs	EG: GSK'872 + CSE Z-VAD + CSE CG: CSE	/	HMGB1, TNF-α, IL-6 (GSK'872) ↓ (Z-VAD) ↑	/
	Female C57BL/6 J mice (aged 6 weeks)	EG: GSK'872 + Smoke Z-VAD + Smoke CG: DMSO + Smoke	p-MLKL(GSK'872) ↓	HMGB1, IL-6 (GSK'872) ↓ Neutrophils, CD8^+^ T cells, macrophage (GSK'872) ↓	Lung chord lengths, alveolar area (GSK'872) ↓
POUWELS S D, 2016	Female BALB/cByJ mice (aged 8 weeks)	EG: Nec-1 + Smoke CG: DMSO + Smoke	/	Neutrophils, MPO ↓	/
MAO K, 2021	Male C57BL/6 mice (aged 8 weeks)	EG: Melatonin + LPS CG: LPS	p-RIPK3, p-MLKL ↓ RIP1, RIP3, MLKL ↓	Airway wall thickness, BALT ↓ cilium number of bronchial epithelial cells↑ IL-1α, IL-1β, TNF-α, ICAM-1, CXCL10, G-CSF ↓ CD4^+^, CD8^+^, CD45^+^ T cells, NK cells, CD11b^+^, CD11c^+^, F4/80^+^ macrophages↓	Alveolar enlargement↓ lung destruction area↓
LU Z, 2021	Acute CS-exposure mice	EG: RIPK3−/− mice CG: WT mice	RIPK3 deletion	Mip1a↓ Total leukocytes, neutrophils, lymphocytes ↓	/
	Acute CS-exposure mice	EG: MLKL^−/−^ mice CG: WT mice	MLKL deletion	Total leukocytes, neutrophils↓ CXCL1, Mmp12, Ym1, Marco↓	/
	Chronic CS-exposure mice	EG: RIPK3^−/−^ mice MLKL^−/−^ mice CG: WT mice	RIPK3 deletion (RIPK3^−/−^) MLKL deletion (MLKL^−/−^)	Total leukocytes, Alveolar macrophage↓ Collagen deposition↓ Ym1↓ Airway inflammation score, Epithelial thickness↓ Marco (RIPK3^−/−^) ↓ lymphocytes (RIPK3^−/−^) ↓ CXCL1, Mmp12, Mmp8 (MLKL^−/−^) ↓ macrophages, neutrophils (MLKL^−/−^) ↓	MLI ↓ Mmp12 (MLKL^−/−^) ↓

↑, The data of the experimental group was significantly higher than that of the control group; ↓, The data of the experimental group was significantly lower than that of the control group; /, There is no relevant expression in this paper; −/−, Gene deletion.

*BALT* bronchus-associated lymphoid tissue, *BEAS-2B* human normal lung epithelial cells, *BMDM* bone marrow–derived macrophages, *CG* control group, *CSE* cigarette smoke extract, *CXCL* CXC chemokine ligand, *DI* destructive index, *DMSO* dimethyl sulfoxide, *EG* experimental group, *G-CSF* granulocyte-colony stimulating factor, *HBE* human bronchial epithelial cells, *HMGB1* high-mobility group box-1, *ICAM-1* circulating intercellular adhesion molecule-1, *IL* interleukin, *LPS* lipopolysaccharide, *MLE-12* mouse lung epithelial cells, *MLI* mean linear intercept, *MLKL* mixed-lineage kinase domain-like, *MPO* myeloperoxidase, *PM* particulate matter, *RIPK* receptor-interacting protein kinase, *TF-3* theaflavin-3,3′-digallate, *TNF* tumour necrosis factor, *WT* wild type.

## Mechanism of necroptosis in COPD

### Abnormal hyperplasia of pro-inflammatory mediators

Lung parenchymal epithelial cells located between environmental stimuli (microorganisms, gases and allergens) and the organism have important functions in maintaining normal respiratory function, including barrier protection, fluid homeostasis, particle clearance, immune response initiation, mucus and surfactant production and post-injury repair. Abnormal lung epithelial cell damage and death are one of the initial events in COPD, as continuous exposure to toxic particles causes an increased incidence of cell death. Necroptosis, an important mode of lung epithelial cell death in COPD, causes the release of cell contents into the extracellular environment, including a large number of danger-associated molecular patterns (DAMPs), such as high-mobility group box-1 (HMGB1), S100 proteins, double-stranded DNA and ATP [30]. The increase in exposure duration and the dose of toxic PM inhibited caspase activity and reduced the transcript levels of *CFLAR* (an encoding gene for transcribing the cell death regulator protein that heterodimerises with caspase-8 to inhibit necroptosis), which induces a gradual switch from apoptosis to necroptosis as the mode of lung epithelial cell death [33–35] and promotes a further increase in the release of DAMPs (including HMGB1, S100A4 and ATP) [25]. After being released into the extracellular environment, DAMPs bind to pattern-recognition receptors on the membranes of neighbouring cells to induce the production of pro-inflammatory cytokines (e.g. C-X-C motif chemokine ligand 8, interleukin [IL]-6) and trigger an immune response in the lung [30].

HMGB1, one of the DAMPs, can be used as a necrosis marker, is highly expressed in the epithelial cells and alveolar macrophages of patients with COPD and is negatively correlated with forced expiratory volume [36, 37]. The receptor for advanced glycation end products (RAGE) is a pattern-recognition receptor [38] and has a high affinity to HMGB1. The number and expression levels of RAGE in bronchiolar epithelial cells, type II alveolar pneumocytes, alveolar macrophages and endothelial cells are low in normal conditions but remarkably increased in COPD [39]. The interaction of HMGB1 with RAGE in the airways of patients with COPD promotes inflammation, fibroblast proliferation, chemotaxis and metalloproteinase synthesis and sequentially leads to tissue remodelling associated with airflow limitation and emphysema, such as airway smooth muscle thickening and fibrosis [37]. As a preparatory step for the IL-1β overproduction induced by ATP from DAMPs, extracellular HMGB1 drives pro-IL-1β generation via the mitogen-activated protein kinase/NF-κB pathway after the activation of the RAGE receptor in macrophages [40]. In addition, the locally secreted HMGB1 binds to IL-1β to form soluble complexes that lead to increased production of the pro-inflammatory cytokine TNF-α in macrophages, amplifying and prolonging the inflammatory and remodelling responses in the airways of smokers [37].

### Insufficient clearance of dead cells

In addition to inducing macrophages to produce large amounts of pro-inflammatory cytokines, necroptosis can recruit macrophages to the lung parenchyma and trigger the phagocytosis of necroptotic epithelial cells by macrophages, but the rate of clearance is slow [8, 25].

During necroptosis, p-MLKL translocation to the cell membrane exposes phosphatidylserine to generate ‘find me’ and ‘eat me’ signals and releases extracellular vesicles containing phosphatidylserine, p-MLKL and other proteins to the surrounding environment, which drive the recognition and uptake of necroptotic cells by macrophages, but the overall phagocytosis efficiency is lower than that of apoptotic cells [41]. Klöditz and Fadeel [42] also indicated that apoptotic cells are more easily phagocytosed by macrophages than necroptotic cells by quantifying phagocytosis efficiency using fluorescent microscopy techniques. However, the ability of alveolar macrophages to phagocytose apoptotic epithelial cells is impaired in COPD [43]. Chen et al. [25] found a remarkable increase in the macrophage uptake of dying MLE-12 cells by a co-culture system of CSE-treated MLE-12 cells with bone marrow–derived macrophages or alveolar macrophages. They observed that the macrophage uptake process can be attenuated by necroptosis inhibition but enhanced by apoptosis inhibition after comparing the experimental groups pre-treated with different pathway inhibitors (GSK’872 or Z-VAD, a caspase inhibitor). These results were likely observed because Z-VAD inhibits apoptosis and provides the conditions for necroptosis to occur. The study also revealed in a real-time video of phagocytosis that dead MLE-12 cells treated with CSE and GSK'872 are more readily and efficiently cleared by macrophages compared to MLE-12 cells treated with CSE and Z-VAD. Thus, necroptotic cells are more easily taken up than apoptotic cells but are more difficult for macrophages to clear under CSE exposure. Necroptosis leads to a massive release of DAMPs (e.g. HMGB1) into the extracellular environment after the plasma membrane rupture of epithelial cells, and the C-terminal acidic tail of HMGB1 is responsible for the inhibition of efferocytosis and binding to RAGE receptors; moreover, HMGB1 can participate in the inhibition process of efferocytosis through interaction with macrophage surface receptors (e.g. RAGE) or phosphatidylserine on apoptotic cell membranes [44, 45]. This finding might explain the less efficient uptake of apoptotic cells by macrophages than necroptotic cells in COPD. However, the slower clearance of necroptotic cells by macrophages along with the abnormal increase in dead non-inflammatory cells causes the inadequate phagocytosis of dead cells, leading to pathological inflammation [46].

### Oxidative stress

Necroptosis and oxidative stress interact with one another, and this circuit may be the autonomous mechanism for the intractable progression of airflow limitation in COPD. On the one hand, key proteins in the necroptosis pathway influence the onset of oxidative stress. The inflammatory mediator TNF-α is remarkably elevated in COPD, and TNF-induced reactive oxygen species (ROS) production is dependent on RIPK3, a key protein in the necroptosis pathway, which may be related to abnormal energy metabolism following activation of the key enzymes of the metabolic pathway by RIPK3 [47]. After the expression levels of RIPK1/RIPK3 are reduced by Nec-1 pre-treatment or RNA interference, ROS production is inhibited in response to TNF stimulation [48]. On the other hand, when a sufficient amount of ROS is present in the cell, this oxidative stress can have enormous effects on the cell, including the induction of cell death [49]. Mitochondrial ROS (mitoROS) modulates the occurrence of necroptosis in COPD, which leads to airflow limitation. Wang et al. [29] specifically inhibited mitoROS in macrophages by mitochondria-targeted antioxidant (Mito-TEMPO) and confirmed that mitoROS mediates CSE-induced necroptosis and inflammatory responses, indicating that the necroptosis pathway is most likely dependent on mitoROS. Xu et al. [9] treated PM-exposed human bronchial epithelial cells by Mito-TEMPO and found that PM exposure produces excessive mitoROS that activates the mitoROS-dependent early growth response gene-1 and therefore mediates the necroptosis of epithelial cells, whereas the mitoROS/early growth response gene-1/necroptosis pathway triggers two distinct signalling cascade responses (NF-κB and AP-1 pathways) to cause airway inflammation and mucus overproduction.

RIPK1/RIPK3-related knockdown and the pharmacological inhibition of RIPK1 by Nec-1 can block ROS production and cell death in response to BV6/TNF-α stimulation, suggesting that RIPK1 and RIPK3 regulate ROS production; by contrast, ROS scavengers can block the interaction between RIPK1 and RIPK3, suggesting that ROS can promote the stabilisation of the RIPK1/RIPK3 necrosome complex to drive necroptosis signalling [50]. Thus, a positive feedback amplification loop exists between RIPK1/RIPK3 and ROS to regulate the BV6/TNF-α–induced necroptosis process. Furthermore, RIPK3-dependent mitoROS induced by TNF can phosphorylate RIPK1 by activating serine residue 161 (a RIPK1 autophosphorylation site) and therefore effectively recruit RIPK3 to form functional necrosomes. This finding also points to the idea that the positive feedback loop consisting of ROS/RIPK1/RIPK3 contributes to the persistence of necroptosis [51].

**Fig. 3:**
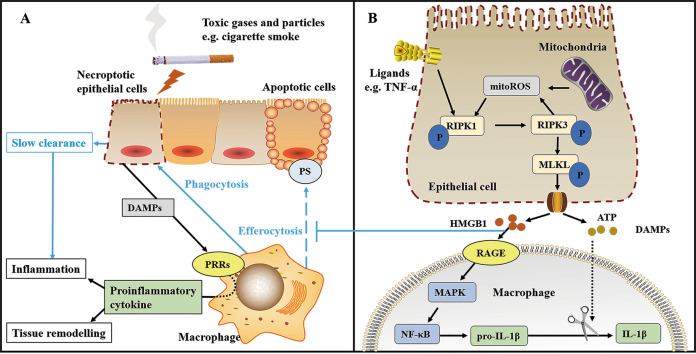
The mechanism of necroptosis leads to airflow limitation in COPD. **A** Lung parenchymal epithelial cells undergo necroptosis due to direct exposure to toxic gases and particles (e.g. cigarette smoke). Excessive DAMPs are released and linked to PRRs on neighbouring macrophages after plasma membrane rupture, leading to pro-inflammatory cytokine production through multiple pathways and consequently causing inflammation and tissue remodelling in the lung parenchyma. In addition, the impaired ability of macrophages to phagocytose apoptotic epithelial cells in COPD and the slower clearance of necroptosis cells by macrophages can cause inadequate phagocytosis of dead cells, which triggers inflammation. **B** Epithelial cell membrane receptors bind to the corresponding ligands (e.g. TNF-α) and send signals to cause RIPK1 activation, followed by RIPK3 recruitment and phosphorylation. Mitochondrial ROS contributes to RIPK1 autophosphorylation, but the production is dependent on RIPK3. RIPK3 can also mediate MLKL phosphorylation, causing MLKL oligomerisation and translocation to the plasma membrane for forming plasma membrane pores and therefore releasing excessive DAMPs (including HMGB1 and ATP) to the extracellular environment. HMGB1 activates RAGE receptors on macrophages and promotes pro–IL-1β production via the MAPK/NF-κB pathway. Pro–IL-1β is cleaved by ATP-induced pathway products to form IL-1β. In addition, HMGB1 interacts with RAGE on the surface of macrophages and PS on the surface of apoptotic cells to participate in the inhibition of the macrophage uptake of apoptotic epithelial cells. ATP adenosine 5′-triphosphate, DAMPs damage-associated molecular patterns, HMGB1 high-mobility group box-1, IL interleukin, MAPK mitogen-activated protein kinase, MitoROS mitochondrial reactive oxygen species, MLKL mixed-lineage kinase domain-like, NF-κB nuclear factor kappa-B, P phosphorylates, PRRs pattern recognition receptors, PS phosphatidylserine, RAGE receptor for advanced glycation end-products, RIPK receptor-interacting protein kinase, TNF tumour necrosis factor.

Figure 3 shows the mechanism of necroptosis leading to airflow limitation in COPD.

## Conclusions

Necroptosis as the primary form of cell death in the late stage of COPD leads to airway inflammation, mucus overproduction, airway remodelling and emphysema and ultimately causes expiratory airflow limitation in COPD. The related mechanism may involve plasma membrane rupture and the release of a large number of DAMPs into the extracellular environment after the necroptosis of lung parenchymal epithelial cells. The released DAMPs activate pattern-recognition receptors in neighbouring macrophages and induce the massive production of pro-inflammatory cytokines that give rise to immune responses and tissue remodelling. In addition, the increased number of necroptotic epithelial cells is difficult for macrophages to rapidly clear, which can also lead to pathological inflammation in the lung. Moreover, a positive feedback loop formed by ROS/RIPK1/RIPK3 exists in the interaction between necroptosis and oxidative stress, further prolonging airflow limitation in COPD. Therefore, studies on how necroptosis affects airflow limitation in COPD are beneficial to understanding the pathological mechanisms of COPD from a new perspective and may provide new ideas for the treatment of progressive airflow limitation in COPD.

## Data Availability

Data sharing is not applicable to this article as no datasets were generated or analysed during the current study.
